# Stepwise Increases in Left Ventricular Mass Index and Decreases in Left Ventricular Ejection Fraction Correspond with the Stages of Chronic Kidney Disease in Diabetes Patients

**DOI:** 10.1155/2012/789325

**Published:** 2011-08-11

**Authors:** Szu-Chia Chen, Jer-Ming Chang, Wan-Chun Liu, Yi-Chun Tsai, Jer-Chia Tsai, Ho-Ming Su, Shang-Jyh Hwang, Hung-Chun Chen

**Affiliations:** ^1^Division of Nephrology, Department of Internal Medicine, Kaohsiung Medical University Hospital, Kaohsiung Medical University, Kaohsiung 807, Taiwan; ^2^Department of Internal Medicine, Kaohsiung Municipal Hsiao-Kang Hospital, Kaohsiung Medical University, Kaohsiung 812, Taiwan; ^3^Department of Renal Care, College of Medicine, Kaohsiung Medical University, Kaohsiung 807, Taiwan; ^4^Division of Cardiology, Department of Internal Medicine, Kaohsiung Medical University Hospital, Kaohsiung Medical University, Kaohsiung 807, Taiwan; ^5^Department of Medicine, College of Medicine, Kaohsiung Medical University, Kaohsiung 807, Taiwan

## Abstract

*Aims*. Patients with diabetic nephropathy are reported to have a high prevalence of left ventricular structural and functional abnormalities. This study was designed to assess the determinants of left ventricular mass index (LVMI) and left ventricular ejection fraction (LVEF) in diabetic patients at various stages of chronic kidney disease (CKD). *Methods*. This cross-sectional study enrolled 285 diabetic patients with CKD stages 3 to 5 from our outpatient department of internal medicine. Clinical and echocardiographic parameters were compared and analyzed. *Results*. We found a significant stepwise increase in LVMI (*P* < 0.001), LVH (*P* < 0.001), and LVEF <55% (*P* = 0.013) and a stepwise decrease in LVEF (*P* = 0.038) corresponding to advance in CKD stages. *Conclusions*. Our findings suggest that increases in LVMI and decreases in LVEF coincide with advances in CKD stages in patients with diabetes.

## 1. Introduction

Diabetic nephropathy is one of the major complications of diabetes mellitus (DM) and one of the major reasons for renal replacement therapy [1]. The leading cause of morbidity and mortality in patients with diabetic nephropathy is cardiovascular disease [2]. Cardiovascular risk in this population can partially be attributed to an increase of traditional risk factors among people with DM but may also be related to the risk factors for coexisting chronic kidney disease (CKD), such as proteinuria, fluid retention, anemia, oxidative stress, and chronic inflammatory state [2–4].

 There are a number of hemodynamic and metabolic disturbances that affect the structure and function of heart in patients with diabetic nephropathy. The major factors that contribute to further heart failure in diabetic patients include cardiac microangiopathy, neuropathy of the cardiac autonomous nervous system, disturbed metabolism, and fatty degeneration of the myocardium [5]. These patients are reported to have a high prevalence of decreased left ventricular systolic function and increased left ventricular mass index (LVMI) resulting from pressure and volume overload [6, 7]. Echocardiographic measures of left ventricular function and structure have been reported to predict adverse cardiovascular outcomes in a variety of populations [8, 9]. Therefore, it is important to detect and treat abnormal geometry and dysfunction of heart early. However, little is known about the relation between the severity of left ventricular geometry and dysfunction and renal function impairment in diabetic patients. The aim of this study was to compare the LVMI and left ventricular ejection fraction (LVEF) among diabetic patients with various degrees of renal insufficiency and identify the independent risk factors associated with increased LVMI and decreased LVEF in this population.

## 2. Subjects and Methods

### 2.1. Study Patients and Design

The study was conducted in a regional hospital in southern Taiwan. In total, 285 diabetic patients with CKD stages 3 to 5 were enrolled consecutively from our outpatient department of internal medicine from January 2007 to May 2010. Patients with evidence of kidney damage lasting for more than 3 months were classified into CKD stage 3, 4, or 5 groups based on estimated glomerular filtration rate (eGFR) level (mL/min/1.73 m^2^) of 30 to 59, 15 to 29, and <15, respectively, as recommended in the National Kidney Foundation-Kidney Disease Outcomes Quality Initiative (K/DOQI) guidelines [10]. Patients with significant mitral valve disease and inadequate image visualization were excluded. The protocol for this study was approved by our institutional review board, and all enrolled patients gave written informed consent.

### 2.2. Evaluation of Cardiac Structure and Function

The echocardiographic examination was performed using VIVID 7 (General Electric Medical Systems, Horten, Norway), with the participant respiring quietly in the left decubitus position. The echocardiographers were blind to patient data. Two-dimensional and two-dimensional guided M-mode images were recorded from the standardized view points. The echocardiographic measurements included aortic root diameter, left atrial diameter (LAD), left ventricular internal diameter in diastole (LVIDd), and left ventricular internal diameter in systole (LVIDs), LVEF, peak early transmitral filling wave velocity (*E*), and peak late transmitral filling wave velocity (*A*). Left ventricular mass was calculated using the Devereux-modified method [11]. LVMI was calculated by dividing left ventricular mass by body surface area. Left ventricular hypertrophy (LVH) was defined when LVMI exceeded 134 g/m^2^ and 110 g/m^2^ for men and women, respectively [12]. Systolic function was assessed by measuring ejection fraction of left ventricle. Systolic dysfunction was defined as LVEF <55%. Diastolic function was estimated by measuring the *E*/*A* ratio; a value of <1.0 was considered diastolic dysfunction.

### 2.3. Collection of Demographic, Medical, and Laboratory Data

Demographic and medical data, including age, gender, smoking history (ever versus never), and comorbid conditions, were garnered from medical records or interviews with patients. Study subjects were defined as having DM if their fasting blood glucose levels were greater than 126 mg/dL or they were taking hypoglycemic agents to control blood glucose levels. Similarly, participants were defined as having hypertension if their systolic blood pressures were ≥140 mmHg or diastolic blood pressure ≥90 mmHg or they were taking antihypertensive drugs. Coronary artery disease was defined if they had a history of typical angina with positive stress test, angiographically documented coronary artery disease, and old myocardial infarction or they had undergone coronary artery bypass surgery or angioplasty. Cerebrovascular disease was defined if they had a history of cerebrovascular incidents such as cerebral bleeding and infarction. Congestive heart failure was defined based on the Framingham criteria. Body mass index was calculated as the ratio of weight in kilograms divided by square of height in meters. Blood and urine samples were obtained within 1 month of enrollment. Laboratory data were measured from fasting blood samples using an autoanalyzer (Roche Diagnostics GmbH, D-68298 Mannheim COBAS Integra 400). Serum creatinine was measured by the compensated Jaffé (kinetic alkaline picrate) method in a Roche/Integra 400 Analyzer (Roche Diagnostics, Mannheim, Germany) using a calibrator traceable to isotope-dilution mass spectrometry [13]. The value of eGFR was calculated using the 4-variable equation in the Modification of Diet in Renal Disease (MDRD) study [14]. The HbA1c was measured by Prismus CLC 385 automated analyzer. Proteinuria was examined by dipsticks (Hema-Combistix, Bayer Diagnostics). A test result of 1+ or more was defined as positive. In addition, information regarding patient medications including aspirin, angiotensin converting enzyme inhibitors (ACEIs), angiotensin II receptor blockers (ARBs), non-ACEI/ARB antihypertensive drugs, and HMG-CoA reductase inhibitors (statins) during the study period was obtained from medical records.

### 2.4. Statistical Analysis

Data are expressed as percentages or mean ± standard deviation or median (25th–75th percentile) for triglyceride. Multiple comparisons among the study groups were performed by one-way analysis of variance (ANOVA) followed by post hoc test adjusted with a LSD correction. The relationship between two continuous variables was assessed by a bivariate correlation method (Pearson's correlation). Linear regression analysis was used to identify the factors associated with LVMI and LVEF. Significant variables in univariate analysis were selected for multivariate analysis. *P* value less than 0.05 was considered significant. All statistical operations were performed using SPSS 12.0 for Windows (SPSS Inc. Chicago, USA).

## 3. Results

**Figure 1:**
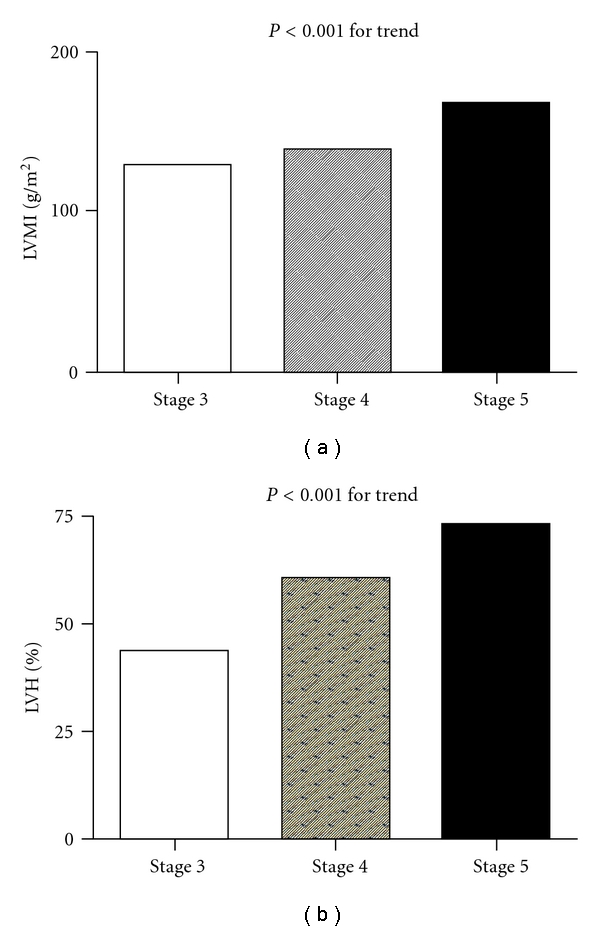
There was a significant trend for a stepwise increase in left ventricular mass index (LVMI) (*P* < 0.001 for trend) (a) and the prevalence of left ventricular hypertrophy (LVH) (44.4%, 61.6%, and 83.9%, resp.; *P* < 0.001 for trend) (b) corresponding to the advancement in chronic kidney disease from stage 3 to 5.

**Figure 2:**
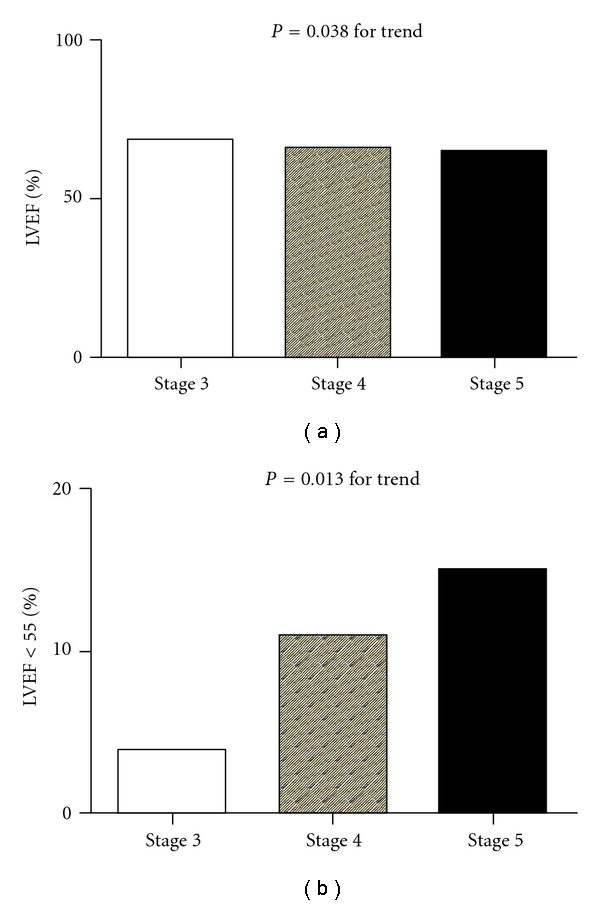
There was a significant trend for a stepwise decrease in left ventricular ejection fraction (LVEF) (*P* = 0.038 for trend) (a) and stepwise increase in the prevalence of LVEF < 55% (4.0%, 11.1%, and 17.2%, resp.; *P* < 0.013 for trend) (b) corresponding to the advancement in chronic kidney disease from stage 3 to 5.

As can be seen in Table 1, a summary of clinical characteristics organized by CKD stage, we studied 285 nondialyzed CKD patients (174 men and 111 women, mean age 66.4 ± 11.6 years). The prevalence of LVH and LVEF < 55% was 62.5% and 10.5%, respectively. Stepwise increases in the prevalence of a history of hypertension, cerebrovascular disease, and congestive heart failure, pulse pressure, uric acid, phosphorous, calcium-phosphorous product, proteinuria, and percentage of non-ACEI/ARB antihypertensive drug use and stepwise decreases in the diastolic blood pressure, albumin, hemoglobin, eGFR, and calcium corresponded to advancement in CKD from stage 3 to 5. In addition, there was a significant trend for a stepwise increase in the LAD, LVIDd, LVIDs, LVMI, and the prevalence of LVH and LVEF < 55% and a stepwise decrease in the LVEF corresponding to advancement in CKD from stage 3 to 5.  Figure 1 shows the significant trend for a stepwise increase in LVMI (a) and the prevalence of LVH (b) corresponding to the advancement in CKD from stage 3 to 5.  Figure 2 shows the significant trend for a stepwise decrease in LVEF (a) and a stepwise increase in the prevalence of LVEF < 55% (b) corresponding to the advancement in CKD from stage 3 to 5.

As seen in Table 2 which summarizes our findings on the possible determinants of LVMI in our study patients, univariate analysis showed a significant positive correlation between LVMI and being male, a history of smoking, coronary artery disease, and congestive heart failure, advanced CKD stages, systolic blood pressure, pulse pressure, phosphorous, proteinuria, aspirin use, and non-ACEI/ARB antihypertensive drug use and negative correlation between LVMI and albumin, hemoglobin, calcium, and ACEI and/or ARB use. Further forward multivariate analysis revealed a significant correlation between increases in LVMI and being male, a history of congestive heart failure, advanced CKD stages, high systolic blood pressure, and low serum albumin level.

Table 3 summarizes the results of our analysis of possible determinants of LVEF in our study patients. Univariate analysis showed a positive correlation between LVEF and albumin, calcium, and ACEI and/or ARB use and a negative correlation with being male, a history of coronary artery disease and congestive heart failure, advanced CKD stages, uric acid, phosphorous, aspirin use, and non-ACEI/ARB antihypertensive drug use. Further forward multivariate analysis revealed a correlation between decreased LVEF and being male, a history of coronary artery disease, advanced CKD stages, low serum albumin level, and ACEI and/or ARB use.

## 4. Discussion

In the present study, we evaluated the determinants of LVMI and LVEF in diabetic patients with various stages of CKD. We found a significant trend for a stepwise increase in LVMI and the prevalence of LVH and LVEF < 55% and a stepwise decrease in LVEF corresponding to advancement in CKD stage.

Patients with diabetic nephropathy have a high prevalence of LVH and left ventricular systolic dysfunction, two disorders that contribute majorly to increased risk of cardiovascular death [2–4]. Structural and functional abnormalities of the heart are common in patients with diabetic nephropathy because of pressure and volume overload [15, 16]. The prevalence of LVH ranges from 17% to 42% in patients with hypertension, 22% to 47% in patients with CKD, and 68.5% of dialysis patients, and LVH occurs in only 3.2% of the general population [8, 17, 18]. However, the prevalence of LVH in our study patients was relatively high (62.5%), which might be explained by the fact that all of the patients included in our study had diabetic nephropathy. The prevalence of left ventricular systolic dysfunction in patients with chronic renal insufficiency is approximately 7.6%–22% [8, 19]. In our patients, the prevalence of LVEF < 55% was 10.5%, which is compatible with previous findings. 

DM, hypertension, and dyslipidemia are traditional cardiovascular risk factors. In addition to these traditional risk factors, patients with CKD may have other risk factors for increase cardiovascular risk such as inflammation, oxidative stress, anemia, metabolic disorders, calcium-phosphorous disorders, hypervolemia, and structural and functional abnormalities of heart, which may help to explain the high cardiovascular morbidity and mortality in such patients [20–25]. LVH, a common finding in patients with CKD, has been reported to advance with decreases in glomerular filtration rate [3]. Hillege et al. found that there was also a significant correlation between the deterioration of congestive heart failure and the progression of renal failure [26]. Our study found that, with the decrease of renal function, there was a significant trend for a stepwise increase in LVMI and the prevalence of LVH and LVEF < 55% and a stepwise decrease in LVEF in patients with diabetic nephropathy, which is consistent with the previous findings.

 Low serum albumin level has been regarded as indicator of malnutrition. Malnutrition may worsen the outcome of CKD by aggravating existing inflammation and heart failure [27]. Hypoalbuminemia has been correlated with left ventricular structure and function [25, 28, 29]. Kursat et al. [25], evaluating the relationship between the degree of malnutrition and echocardiographic parameters in 72 hemodialysis patients, found that the malnutrition index, calculated using Subjective Global Assessment, had a positive correlation with left ventricular mass and index. They cited inadequate volume control as an explanation for their findings. Volume overload may substantially decrease energy and protein intake, suggesting a possible relation between volume overload and malnutrition. In addition, volume overload may increase the diastolic wall stress and in turn cause the development of LVH [25]. Trovato et al. [29], also investigating the correlation between heart failure and nutritional status in hemodialysis patients, reported an association between low serum albumin level and decreased LVEF. Our results consistently demonstrate independent association between low serum albumin levels and increased LVMI and decreased LVEF in patients with diabetic nephropathy.

One limitation of this study was that it had a cross-sectional design, and thus the predictors of cardiovascular events could not be evaluated. Further prospective studies are needed to confirm our findings.

In conclusion, our results found a significant trend for a stepwise increase in LVMI and the prevalence of LVH and LVEF < 55% and a stepwise decrease in LVEF corresponding to advancement in CKD stage in diabetic patients.

##  Disclosure

The authors have no financial interest in the information contained in the paper. 

## Figures and Tables

**Table 1:** Clinical characteristics of patients among different stages of CKD.

Characteristics	Stage 3 (*n* = 99)	Stage 4 (*n* = 99)	Stage 5 (*n* = 87)	*P* for trend	All patients (*n* = 285)
Age (year)	66.3 ± 12.4	68.4 ± 10.7	64.1 ± 11.5^†^	0.039	66.4 ± 11.6
Male gender (%)	75.8	58.6*	47.1*	<0.001	61.1
Smoking history (%)	32.3	36.4	28.7	0.540	32.6
Hypertension (%)	79.8	81.8	97.7^∗†^	0.001	86.0
Coronary artery disease (%)	13.1	13.1	18.4	0.514	14.7
Cerebrovascular disease (%)	10.1	21.2*	27.6*	0.009	19.3
Congestive heart failure (%)	10.1	15.2	28.7^∗†^	0.003	17.5
Systolic blood pressure (mmHg)	144.5 ± 21.2	141.2 ± 20.0	148.3 ± 23.7^†^	0.089	144.6 ± 21.8
Diastolic blood pressure (mmHg)	82.4 ± 12.3	77.4 ± 11.9*	76.7 ± 14.1*	0.005	78.9 ± 13.0
Pulse pressure (mmHg)	62.1 ± 16.7	63.8 ± 11.7	71.7 ± 19.9^∗†^	0.001	65.7 ± 18.2
Body mass index (kg/m^2^)	26.2 ± 4.0	26.3 ± 3.5	25.0 ± 3.8^∗†^	0.043	25.9 ± 3.8

Laboratory parameters					
Albumin (g/L)	41.3 ± 3.5	39.9 ± 3.9*	37.3 ± 4.5^∗†^	<0.001	39.6 ± 4.3
Fasting glucose (mmol/L)	8.2 ± 3.2	8.0 ± 3.8	8.3 ± 4.7	0.896	8.1 ± 3.9
HbA1c (%)	7.5 ± 1.4	8.1 ± 2.1*	7.4 ± 1.8^†^	0.032	7.7 ± 1.8
Triglyceride (mmol/L)	1.8 (1.1–2.4)	1.8 (1.4–2.6)	1.8 (1.2–2.7)	0.173	1.8 (1.2–2.6)
Total cholesterol (mmol/L)	5.0 ± 1.1	5.1 ± 1.3	5.2 ± 1.4	0.544	5.1 ± 1.3
Hemoglobin (g/L)	128.8 ± 18.7	115.4 ± 19.2*	93.1 ± 13.4^∗†^	<0.001	113.2 ± 22.7
Baseline eGFR (mL/min/1.73 m^2^)	40.5 ± 6.6	23.1 ± 4.5*	10.3 ± 3.0^∗†^	<0.001	25.2 ± 13.3
Calcium (mmol/L)	2.4 ± 0.2	2.4 ± 0.2	2.3 ± 0.2^∗†^	<0.001	2.4 ± 0.2
Phosphate (mmol/L)	1.1 ± 0.2	1.3 ± 0.2*	1.6 ± 0.4^∗†^	<0.001	1.3 ± 0.3
Calcium-phosphorous product (mmol^2^/L^2^)	2.8 ± 0.5	3.1 ± 0.6*	3.6 ± 0.8^∗†^	<0.001	3.1 ± 0.7
Uric acid (*μ*mol/L)	456.7 ± 113.3	505.4 ± 138.6*	530.8 ± 143.3*	0.001	496.5 ± 135.1
Proteinuria (%)	47.5	75.5*	98.9^∗†^	<0.001	72.9

Medications					
Aspirin use (%)	30.2	32.3	34.5	0.826	32.2
ACEI and/or ARB use (%)	80.2	83.3	63.1^∗†^	0.003	76.1
Non-ACEI/ARB antihypertensive drug use (%)	67.7	80.8*	94.3^∗†^	<0.001	80.4
Statin use (%)	36.5	29.2	31.0	0.532	32.2

Echocardiographic data					
Aortic root diameter (cm)	3.3 ± 0.4	3.3 ± 0.4	3.2 ± 0.4	0.262	3.3 ± 0.4
LAD (cm)	3.7 ± 0.6	3.9 ± 0.6	4.1 ± 0.6^∗†^	< 0.001	3.9 ± 0.6
LVIDd (cm)	4.8 ± 0.7	4.9 ± 0.8	5.1 ± 0.7^∗†^	0.005	4.9 ± 0.8
LVIDs (cm)	2.9 ± 0.7	3.1 ± 0.8	3.3 ± 0.8^∗†^	0.002	3.1 ± 0.8
LVMI (g/m^2^)	129.5 ± 43.5	139.1 ± 52.4	167.1 ± 45.9^∗†^	<0.001	144.3 ± 49.8
LVH (%)	44.4	61.6*	83.9^∗†^	<0.001	62.5
LVEF (%)	69.0 ± 11.1	67.0 ± 11.7	64.5 ± 13.2*	0.038	66.9 ± 12.1
LVEF < 55% (%)	4.0	11.1*	17.2*	0.013	10.5
*E*/*A* < 1 (%)	78.9	84.9	75.0	0.250	79.8

CKD: chronic kidney disease; eGFR: estimated glomerular filtration rate; ACEI: angiotensin converting enzyme inhibitor; ARB: angiotensin II receptor blocker; LAD: left atrial diameter; LVIDd: left ventricular internal diameter in diastole; LVIDs: left ventricular internal diameter in systole; LVMI: left ventricular mass index; LVH: left ventricular hypertrophy; LVEF: left ventricular ejection fraction; E: peak early transmitral filling wave velocity; A: peak late transmitral filling wave velocity.

*P < 0.05 compared to stage 3; ^†^ *P* < 0.05 compared to stage 4.

**Table 2:** Determinants of left ventricular mass index (LVMI) in study patients.

Characteristics	Univariate		Multivariate (forward)	
	Standardized coefficient *β*	*P*	Standardized coefficient *β*	*P*
Age (year)	0.012	0.842	—	—
Male *versus* female	0.117	0.048	0.211	<0.001
Smoking(ever *versus* never)	0.126	0.033	—	—
Coronary artery disease	0.147	0.013	—	—
Cerebrovascular disease	0.024	0.681	—	—
Congestive heart failure	0.262	<0.001	0.196	0.001
CKD stage	0.301	<0.001	0.262	<0.001
Systolic blood pressure (mmHg)	0.225	<0.001	0.203	<0.001
Diastolic blood pressure (mmHg)	0.095	0.118	—	—
Pulse pressure (mmHg)	0.202	0.001	—	—
Body mass index (kg/m^2^)	0.049	0.413	—	—

Laboratory parameters				
Albumin (g/L)	−0.321	<0.001	−0.132	0.032
Fasting glucose (mmol/L)	0.027	0.653	—	—
HbA1c (%)	−0.052	0.389	—	—
Triglyceride (Log mmol/L)	−0.034	0.570	—	—
Cholesterol (mmol/L)	0.047	0.436	—	—
Hemoglobin (g/L)	−0.212	<0.001	—	—
Calcium (mmol/L)	−0.185	0.002	—	—
Phosphate (mmol/L)	0.182	0.002	—	—
Calcium-phosphorous product (mmol^2^/L^2^)	0.114	0.060	—	—
Uric acid (*μ*mol/L)	0.117	0.052	—	—
Proteinuria	0.216	<0.001	—	—

Medications			—	—
Aspirin use (%)	0.190	0.001	—	—
ACEI and/or ARB use (%)	−0.167	0.005	—	—
Non-ACEI and/or ARB antihypertensive drug use (%)	0.188	0.001	—	—
Statin use (%)	−0.041	0.502	—	—

Values expressed as standardized coefficient *β*. Abbreviations are the same as Table 1.

**Table 3:** Determinants of left ventricular ejection fraction (LVEF) in study patients.

Characteristics	Univariate		Multivariate (forward)	
	Standardized coefficient *β*	*P*	Standardized coefficient *β*	*P*
Age (year)	0.062	0.297	—	—
Male *versus* female	−0.223	<0.001	−0.227	<0.001
Smoking(ever *versus* never)	−0.090	0.130	—	—
Coronary artery disease	−0.169	0.004	−0.153	0.008
Cerebrovascular disease	−0.071	0.235	—	—
Congestive heart failure	−0.155	0.009	—	—
CKD stage	−0.151	0.011	−0.173	0.007
Systolic blood pressure (mmHg)	−0.038	0.528	—	—
Diastolic blood pressure (mmHg)	−0.092	0.130	—	—
Pulse pressure (mmHg)	0.020	0.747	—	—
Body mass index (kg/m^2^)	0.010	0.871	—	—

Laboratory parameters				
Albumin (g/L)	0.258	< 0.001	0.188	0.003
Fasting glucose (mmol/L)	−0.097	0.107	—	—
HbA1c (%)	−0.040	0.509		
Triglyceride (Log mmol/L)	−0.039	0.518	—	—
Cholesterol (mmol/L)	−0.069	0.247	—	—
Hemoglobin (g/L)	0.103	0.083	—	—
Calcium (mmol/L)	0.122	0.044		
Phosphate (mmol/L)	−0.168	0.005		
Calcium-phosphorous product (mmol^2^/L^2^)	−0.103	0.088	—	—
Uric acid (*μ*mol/L)	−0.146	0.015	—	—
Proteinuria	−0.096	0.106	—	—

Medications				
Aspirin use (%)	−0.132	0.028	—	—
ACEI and/or ARB use (%)	0.203	0.001	0.143	0.014
Non-ACEI and/or ARB antihypertensive drug use (%)	0.048	0.422	—	—
Statin use (%)	0.004	0.951	—	—

Values expressed as standardized coefficient *β*. Abbreviations are the same as Table 1.
